# The Effect of an Intervening Promoter Nucleosome on Gene Expression

**DOI:** 10.1371/journal.pone.0063072

**Published:** 2013-05-20

**Authors:** Rasesh Y. Parikh, Harold D. Kim

**Affiliations:** School of Physics, Georgia Institute of Technology, Atlanta, Georgia, United States of America; George Mason University, United States of America

## Abstract

Nucleosomes, which are the basic packaging units of chromatin, are stably positioned in promoters upstream of most stress-inducible genes. These promoter nucleosomes are generally thought to repress gene expression due to exclusion; they prevent transcription factors from accessing their target sites on the DNA. However, the role of promoter nucleosomes that do not directly occlude transcription factor binding sites is not obvious. Here, we varied the stability of a non-occluding nucleosome positioned between a transcription factor binding site and the TATA box region in an inducible yeast promoter and measured downstream gene expression level. We found that gene expression level depends on the occupancy of the non-occluding nucleosome in a non-monotonic manner. We postulated that a non-occluding nucleosome can serve both as a vehicle of and a barrier to chromatin remodeling activity and built a quantitative, nonequilibrium model to explain the observed nontrivial effect of the intervening nucleosome. Our work sheds light on the dual role of nucleosome as a repressor and an activator and expands the standard model of gene expression to include irreversible promoter chromatin transitions.

## Introduction

The effect of promoter nucleosomes is generally thought to be repressive for gene expression because nucleosomes can sterically hinder access of transcription factors to the promoter during transcriptional activation [1]. Genome-wide data show that transcript levels tend to decrease with increasing promoter nucleosome occupancy to some degree, either across many genes in one condition or for one gene across many different conditions [2], [3]. The causal relationship between the two has also been investigated. Increasing transcriptional activity has been found to cause nucleosome occupancy at promoters to decrease [4]. However, depletion of histones does not cause increased expression for all genes, but decreased expression for some [5], [6]. These results suggest that the effect of nucleosomes on gene expression level might vary from promoter to promoter in a nontrivial manner, and call for in-depth studies at smaller scales [7].

**Figure 1:**
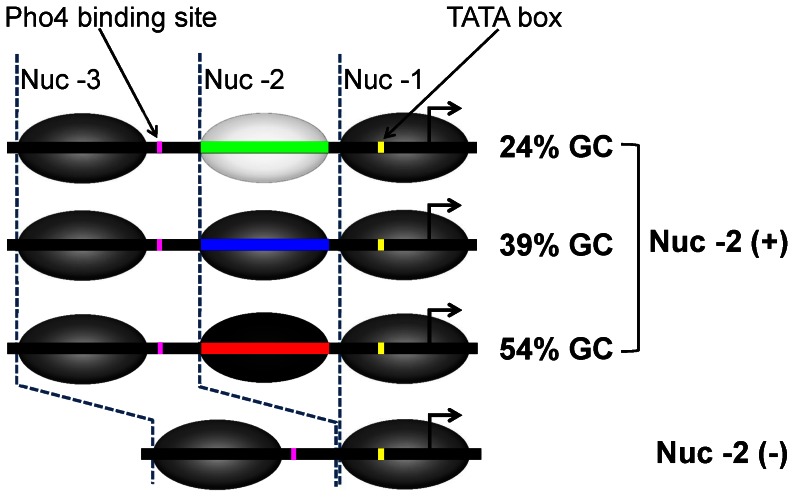
Chromatin architecture of the promoter variants used in this study. DNA is shown as a solid black line. Nucleosomes -1, -2 and -3 are numbered relative to the transcription start site (TSS) and shown in dark ellipses. The direction of transcription is indicated by the black bent arrow. The TATA box is marked in nucleosome -1 (solid yellow tick). A high affinity Pho4 binding site (CACGTG) is located in the linker region between nucleosome -3 and nucleosome -2 (solid purple tick). Nucleosome -2 is the non-occluding nucleosome. Three Nuc -2 (+) variants are used in this study: low (24%), intermediate (39%), and high (54%) GC% DNA sequence for the non-occluding nucleosome. This DNA segment is deleted in Nuc -2 (−) promoter variant.

The PHO5 promoter (PHO5pr) of *Saccharomyces cerevisiae* has been used extensively to study nucleosome remodeling during transcriptional activation [8]–[13] and to build quantitative models of eukaryotic gene expression [14]–[16]. In high phosphate conditions, the transcription factor Pho4 is phosphorylated and cytoplasmic [17], and PHO5pr is repressed with stably positioned nucleosomes -3, -2 and -1 (Figure 1). A low affinity Pho4 binding site is located between nucleosomes -3 and -2, a high affinity Pho4 binding site under nucleosome -2, and the TATA box (the assembly site of general transcription machinery) under nucleosome -1 [18]–[20]. In low phosphate conditions, Pho4 becomes dephosphorylated and nuclear [17] and recruits chromatin remodeling complexes to PHO5pr [21], [22]. As a result, nucleosomes are removed from the promoter, and general transcription factors (GTF) assembled over the exposed TATA box region to initiate transcription [10], [11].

When the low affinity site in the exposed region is ablated, PHO5pr activity is significantly lowered, suggesting that removal of nucleosome -2 is initiated by binding of Pho4 to the exposed binding site, and the high affinity binding site in nucleosome -2 remains inaccessible [19], [20], [23]. Similarly, the TATA box is occluded by nucleosome -1 as the removal of nucleosome -1 is sufficient for transcriptional activation of PHO5pr [24]. These evidences point to the notion that promoter nucleosomes in occluding configurations repress gene expression by preventing trans-factors from binding to cis-regulatory elements. Detailed investigation of this repressive effect of occluding nucleosomes has been recently reported [25].

However, it is not obvious how nucleosomes would affect gene expression if they are not positioned over known regulatory sequences. This question bears physiological relevance because some promoter nucleosomes are found in non-occluding configurations [26], [27]. To shed light upon the role of non-occluding promoter nucleosomes, we constructed a promoter, slightly modified from PHO5pr, which has a non-occluding nucleosome intervening between a high affinity Pho4 binding site and the TATA box. This promoter variant was previously shown to be highly inducible in a Pho4-dependent manner [15], [20], [28]. We changed the occupancy of this nucleosome by changing GC% of the underlying DNA sequence to study its effect on gene expression. We measured the gene regulation function (GRF), which is the relationship between transcription factor input and gene expression output [15], with varying nucleosome occupancy, and found that the non-occluding nucleosome does not always exert a repressive effect on gene expression. To explain the non-monotonic effect of nucleosome occupancy on gene expression level, we developed a nonequilibrium model based on the mechanism of nucleosome removal where the chromatin remodeling complex binds one nucleosome to remove a neighboring nucleosome. Our model also uses self-squelching mechanism to account for saturation of gene expression level.

## Results

For this study, we designed a synthetic promoter with a non-occluding nucleosome based on the PHO5pr in budding yeast. The wild-type PHO5pr has a low affinity Pho4 binding site in the nucleosome depleted region (NDR), a high affinity Pho4 binding site under the intervening nucleosome (nucleosome -2), and the TATA box under nucleosome -1. Thus, the intervening nucleosome in the wild-type promoter architecture occludes a transcription factor binding site. We deleted the high affinity site from nucleosome -2 to change this nucleosome to a non-occluding one and replaced the low affinity site in the NDR with a high affinity site to make the promoter highly inducible (Figure 1). It has been shown that these modifications do not alter positioning or occupancy of PHO5pr nucleosomes [20]. We termed this promoter variant “Nuc -2 (+)”.

To investigate the effect of this non-occluding nucleosome on gene expression level, we constructed two more Nuc -2 (+) variants with different nucleosome -2 stability. The intrinsic DNA sequence is one of the most important factors influencing nucleosome occupancy in vivo [1], [29], and in recent studies, the percentage of G and C in DNA (hereafter referred to as GC%) has emerged as the dominant parameter that governs intrinsic preference for nucleosome formation [30], [31]. Motivated by these studies, we varied the GC% of the 147-bp DNA in the nucleosome -2 region to alter the stability of nucleosome -2. The nucleosome -2 sequence, equivalent to the wild-type PHO5pr except for the absence of the high affinity binding site, was 39% in GC content. Using this sequence as the reference sequence for intermediate GC%, we generated higher GC% sequences by randomly replacing A or T with G or C, and lower GC% sequences by randomly replacing G or C with A or T. Among these sequences, we chose two that are least perturbed in terms of additional transcription factor binding sites, one with higher GC% (54%) and another with lower GC% (24%) than the intermediate GC% sequence.

**Figure 2:**
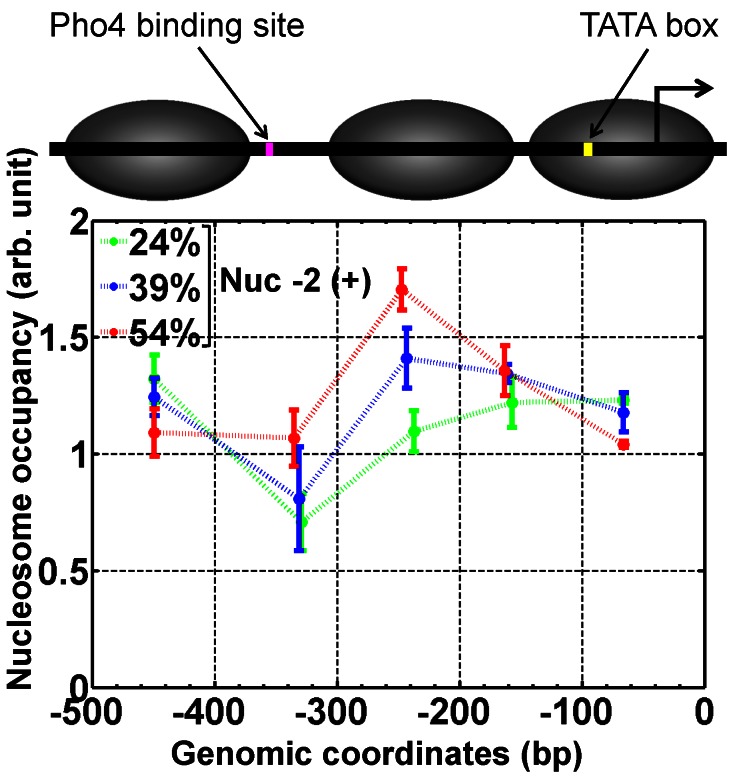
In-vivo nucleosome occupancy maps of Nuc -2 (+) variants. Relative nucleosome occupancies (in arbitrary units) were measured by quantitative ChIP at five locations (in base pairs) which are near or in between the putative centers of promoter nucleosomes according to the compiled data base for nucleosome positioning in *S. cerevisiae* [33]. The nucleosome occupancy at REC104 locus was used as the reference value. The error bars represent the standard deviation of three separate ChIP measurements starting with independent sample preparation.

We compared the occupancy of nucleosome -2 of these Nuc -2 (+) variants under repressive condition using chromatin immunoprecipitation (ChIP). In addition to the occupancy of nucleosome -2, we also measured occupancy of nucleosomes -1 and -3 to ensure that changing the DNA sequence in the nucleosome -2 region did not perturb the position of the neighboring nucleosomes. As expected, nucleosome -2 occupancy increased with the GC% as shown by the ChIP signal at the putative center of nucleosome -2 in Figure 2. In contrast, modification of nucleosome -2 sequence did not affect either position or occupancy of nucleosome -1 and -3. Nucleosome occupancy at the linker regions also increased with higher GC%, which we interpreted as a spillover effect due to the increased nucleosome -2 occupancy.

We then measured the GRFs of Nuc -2 (+) variants, using a doxycycline inducible strain as described in our previous study [15]. Briefly, the transcription factor Pho4 was placed under the control of a doxycycline inducible promoter TETO7pr, and was genetically modified and tagged with yellow fluorescent protein (YFP) so that Pho4 level inside the nucleus could be varied with doxycycline and measured by fluorescence microscopy. Cyan fluorescent protein (CFP) was placed under the control of PHO5pr to report the promoter activity. This way, YFP and CFP intensities could reflect the input and output of the GRF, respectively.

**Figure 3:**
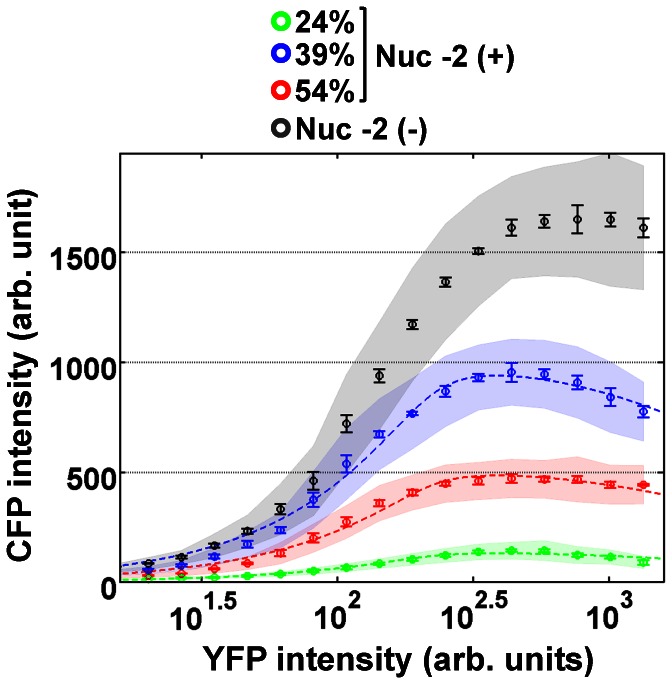
Gene regulation functions of Nuc -2 (+) and Nuc -2 (−) variants. To generate the GRF, data points were binned by their YFP (input) intensity values, and the mean and standard deviation of CFP (output) intensity values within each bin were obtained. The mean and standard deviation from each bin were averaged from three independent measurements. Double-averaged CFP intensities are shown as circles, and the averaged standard deviations are indicated by the vertical width of the shaded region. The error bar represents the standard error of the mean. Nuc -2 (**−**) variant is shown in grey, and 24%, 39%, and 54% Nuc -2 (+) variants are shown in green, blue, and red, respectively. The GRFs of Nuc -2 (+) variants are fitted to Equation 1. p_1_ = 10^2.4^, p_2_ = 10^3.6^, p_4_ = 0.97, and p_3_ is 99, 703, and 363 for 24, 39, and 54 GC%, respectively.

Interestingly, we found a non-monotonic relationship between GRF amplitude and the occupancy of the non-occluding nucleosome (Figure 3). Unlike an occluding nucleosome whose depletion would lead to increased gene expression level, the lowest GC% of nucleosome -2 did not correspond to the highest GRF amplitude. Instead, the amplitude of the GRF was the lowest at the low GC% and the highest at the intermediate GC%. We also noticed that all three GRFs dropped after they reached their maximum. Further increase in Pho4 concentration beyond this point led to severe growth defects as reported [32], and was thus avoided.

Because changing GC% of nucleosome -2 sequence which was similar to the wild-type sequence led to a drop in gene expression level, we explored the possibility that crucial cis regulatory elements could have been unintentionally deleted in the low or high GC% Nuc -2 (+) variant. We constructed another promoter variant which lacks the entire nucleosome -2 region (Nuc -2 (**−**)) by deleting 161-bp long DNA that spans nucleosome -2 and the linker region between nucleosome -2 and -1 [33]. We measured nucleosome positions of this variant, and showed that deletion of nucleosome -2 did not change the occupancy of either nucleosome -3 or -1 (Figure S1). The Nuc -2 (−) showed a higher GRF amplitude than all three Nuc -2 (+) variants, almost 1.5-fold higher than the intermediate GC% Nuc -2 (+). Moreover, all known binding sequences of Pho2 [34], a trans-activator which cooperatively interacts with Pho4, were left intact in all Nuc -2 (+) promoter variants. Based on these results, we believe that this nontrivial effect of the non-occluding nucleosome on gene expression level is a result of nucleosome occupancy, not the change in DNA sequence itself.

## Discussion

We showed that gene expression level depended on the occupancy of a non-occluding nucleosome in a non-monotonic manner. When the nucleosome occupancy was elevated compared to the intermediate level, the gene expression level decreased. This negative effect of nucleosome occupancy on gene expression level was also observed for an intervening nucleosome in GAL1-10 promoter [35]. Surprisingly, however, when we lowered nucleosome occupancy below the intermediate level, the gene expression level decreased, indicating that a non-occluding nucleosome is sometimes indispensable. This positive effect of nucleosomes on gene expression is not intuitive at first and thus is worthy of further consideration.

**Figure 4:**
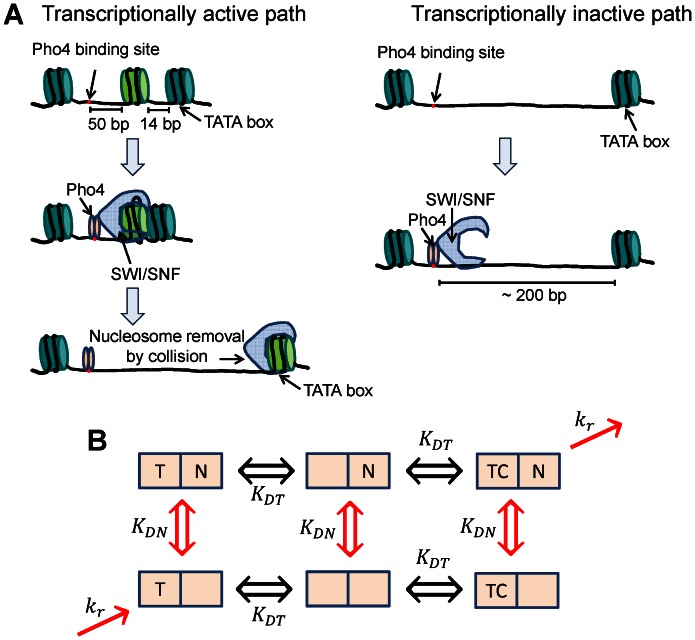
Modeling the effect of the intervening promoter nucleosome on gene expression. (A) The proposed model for nucleosome removal by the chromatin remodeling complex. The promoter is in dynamic equilibrium between two states based on the occupancy of nucleosome -2. The chromatin remodeling complex SWI/SNF is recruited by the transcription factor Pho4 to the linker region. If nucleosome -2 is present (transcriptionally active path), SWI/SNF can bind it and begin pulling DNA within nucleosome -2 from nucleosome -1. This results in collision between nucleosome -1 and the head of SWI/SNF, and the eventual removal of nucleosome -1. If nucleosome -2, however, is not present (transcriptionally inactive path), SWI/SNF cannot easily reach the next nearest nucleosome -1 because of ∼200 bp distance. Participating molecules are drawn approximately to scale. (B) Near-equilibrium model of gene expression. The letters, T, C, and N represent transcription factor, nucleosome, and chromatin remodeling complex, respectively. The promoter state is defined by the occupancy of T, C, and N. The first square represents the transcription factor binding site, and the second square the position for the non-occluding nucleosome. *K_DT_ is* the equilibrium dissociation constant for T⋅DNA T+DNA, and *K_DN_* for N⋅DNA N+DNA. *K_DN_* represents nucleosome stability and varies among Nuc -2 (+) variants. The irreversible chromatin remodeling step represented by *k_r_* brings (1,1,1) back to (1,0,0), and completes the steady-state cycle.

We speculate that a nucleosome can positively contribute to gene expression because chromatin remodeling complex SWI/SNF uses a nucleosome as a substrate for chromatin remodeling [36]. Moreover, it was shown in vitro that SWI/SNF cannot remove a nucleosome from DNA in a mononucleosome context, but can do so in a dinucleosome context [37]. Based on this finding, a model for nucleosome removal was proposed in which SWI/SNF needs to bind one nucleosome to remove another. A similar mechanism was also proposed by Boeger and Kornberg based on in vivo data [14]. We highlight this mechanism in the context of PHO5pr in Figure 4A. When SWI/SNF is recruited to the promoter via Pho4, it can bind nucleosome -2 if available (transcriptionally active path). After binding nucleosome -2 SWI/SNF will translocate DNA within nucleosome -2, which results in shortening of the linker DNA between nucleosome -2 and nucleosome -1, and eventual removal of nucleosome -1. If nucleosome -2 is not present (transcriptionally inactive path), “catching” of nucleosome -1 by SWI/SNF will be inefficient because of the ∼200-bp gap, which is only slightly longer than the persistence length of DNA.

Based on this reasoning, we assume that the promoter switches between two states with and without the intervening nucleosome -2. The chromatin remodeling complex can move the promoter state forward into a transcriptionally active state when nucleosome -2 is present, but is stalled when it is absent. In this view, nucleosomes are not bound 100% of the time, but can continuously assemble and disassemble in equilibrium. This view is seemingly inconsistent with most in vitro experiments where nucleosomes appear to be stable with the half-life of ∼0.6–4 hours [38]. However, nucleosomes, even on extremely strong positioning sequences such as the Widom 601 sequence, are shown to adopt intermediate conformations where the interface between H2A–H2B dimer and (H3-H4)_2_ tetramer is open or where some histones are missing [39], [40]. Thus, the population of these partially open states might be much more significant for regular DNA sequences and chemically modified histones in vivo. Interestingly, in support of this idea, even well-positioned nucleosomes are shown to have less than 50% probability of occupying a region at any given time [35], [41]. Here we assume that these partially open states constitute the nucleosome-unbound fraction which does not become transcriptionally active. Using the equilibrium constant *K_DN_* for the stability of the intervening nucleosome (lower *K_DN_* corresponding to higher stability), the nucleosome-bound fraction becomes 1/(*K_DN_* +1).

On the other hand, nucleosome stability can generally impact gene expression in a negative manner because the remodeling rate will be limited by how frequently the contacts between DNA and the histone octamer core are broken. One can incorporate nucleosome stability into the remodeling rate by assuming that SWI/SNF uses ATP to bias the spontaneous DNA unwrapping/wrapping equilibrium of a nucleosome like a Brownian ratchet [42], [43]. In this model, when DNA unwraps from the histone octamer near one end of a nucleosome, SWI/SNF can translocate in an ATP-dependent manner, and bias the rewrapping location of the DNA. The transiently formed loop will propagate around the histone octamer, resulting in a net displacement of the nucleosome [44]. Hence, we assume a Michaelis-Menten like relationship between the remodeling rate and nucleosome stability where the remodeling rate (*k_r_*) is a logistic function of *K_DN_*.

For all three GRFs with different nucleosome occupancy, we observed that the gene expression level increased sigmoidally with increasing transcription factor level and gradually decreased after reaching the maximum level. Although the exact mechanism remains to be investigated, we speculate that titration of chromatin remodeling complexes by the transcription factor might cause this concave-down GRF at high transcription factor input level. The key idea is that binding of Pho4 alone to PHO5pr does not lead to efficient nucleosome removal. Hence, when Pho4 level is low, most of them will be taken up by SWI/SNF, and Pho4-SWI/SNF complex can bind to PHO5pr to remove nucleosomes. However, when Pho4 level is higher than that of SWI/SNF, free Pho4 will compete with Pho4-SWI/SNF for binding to PHO5pr. Effectively, free Pho4 (T) acts as a repressor, and the promoter fraction that is bound by Pho4-SWI/SNF (TC) is given by [*TC*]/([*T*]+[*TC*]+*K_DT_*) where *K_DT_* is the equilibrium dissociation constant of transcription factor binding to DNA, and ‘[]’ denotes concentration. This hypothesis is similar to the squelching mechanism demonstrated by Gill and Ptashne [45] in that an activator can affect gene expression negatively, but is also clearly different in that an activator represses expression of its own target gene at high levels.

The positive effect of nucleosome occupancy on gene expression and saturation of gene expression can be explained by an equilibrium model which associates TC-bound nucleosome -2 with high transcriptional activity [46]. However, the negative effect of nucleosome occupancy on gene expression has to be incorporated into an irreversible chromatin remodeling rate. To include all these effects in one consistent quantitative framework, we built a non-equilibrium model of gene expression, the details of which are presented in the supporting information (Text S1, Figure S2 and Table S1). In this model, transcriptional activity is proportional to the frequency of ATP-dependent nucleosome removal which is equivalent to a steady-state flux instead of a steady-state probability. The steady-state solution of this flux can be obtained, but is very lengthy. Here, we present an analytical function that describes the steady-state flux near-equilibrium. If the nucleosome removal (*k_r_*) is very slow compared to other reaction steps, one can use the pre-equilibrium assumption for all reversible reactions (Figure 4B), and the flux can be parameterized with four fitting coefficients:
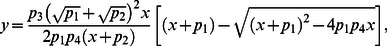
(1)


We performed global fitting of the measured GRFs to Equation 1. The fitting parameters p_1_, p_2_, p_3_, and p_4_ represent the rise threshold, the fall threshold, the maximum level, and the skewness, which can be conveniently estimated from the GRF (Table S2). Because p_1_, p_2_, and p_4_ are independent of nucleosome stability, we allowed only p_3_ to vary among the promoter variants while constraining p_1_, p_2_ and p_4_. The results of this global fitting are shown in Figure 3. Although Equation 1 is obtained near equilibrium, it can also well describe the steady state flux out of equilibrium up to a certain extent (Figure S5). In general, Equation 1 is better suited to fitting the unique features of the measured GRF than the popular Hill equation (Figure S3). We attempted to fit each GRF independently using Equation 1 as a purely phenomenological function, the result of which is presented in Figure S4.

The higher GRF amplitude of Nuc -2 (**−**) than Nuc -2 (+) variants can be due to multiple reasons. First, removing two nucleosomes in a row might be significantly slower than removing one nucleosome. Second, the chromatin remodeling complex is not completely processive, thus having a higher failure rate to remove the second nucleosome than the first. Third, Pho4 might be able to directly stabilize the general transcription machinery and increase the transcription rate only in Nuc -2 (**−**) due to proximity [16]. We expect not only nucleosome -2, but also nucleosome -1 to undergo assembly and disassembly in equilibrium based on similar ChIP amplitudes (Figure S1), which would lead to intermittent exposure of the TATA box even in the absence of Pho4. However, gene expression level of Nuc -2 (**−**) was as negligible as that of any Nuc -2 (+) in the absence of Pho4. Measuring absolute occupancy of nucleosome -1 and TATA binding proteins will shed light on this remaining question.

In summary, our work revealed a nontrivial effect of the non-occluding nucleosome on gene expression level. The effect of the non-occluding nucleosome was assessed by two different approaches: (1) alteration of nucleosome stability by changing the GC% of the underlying DNA sequence and (2) deletion of the underlying DNA. We found that destabilization of the non-occluding nucleosome by lowering the GC% from the wild-type level led to lower gene expression level. This observation is consistent with a nucleosome removal model where the chromatin remodeling complex needs to bind a nucleosome to remove other nucleosomes. Stabilization of the nucleosome also led to lower gene expression level, which raises the possibility that wild-type nucleosome DNA sequences might be evolutionarily optimized for maximum gene expression level. The obvious next step will be to correlate absolute occupancy levels of promoter nucleosomes with gene expression level genome-wide to gain further insights into the role of nucleosomes in gene regulation.

## Materials and Methods

### Yeast strains

All strains for GRF measurement were prepared first by generating a test strain and mating it with the base strain that contained the transcriptional circuits required for doxycycline induction of Pho4 expression and the nuclear marker as previously published [15]. To generate, the PHO5 promoter variant strains, a 161-bp sequence containing nucleosome -2 in the native PHO5pr was either deleted or replaced with a target GC% sequence following a two-step homologous recombination protocol Delitto perfetto [47]. The native high affinity Pho4 binding site in nucleosome -2 was ablated [20], and the native low affinity Pho4 binding sequence between nucleosome -3 and nucleosome -2 was switched to a high affinity one during this procedure. We then replaced PHO5 ORF with Cerulean [48] in all strains using homologous recombination and mated them with the base strain. After sporulation, tetrads were dissected, and haploid cells containing all genetic markers were selected by sequential replica plating onto appropriate dropout or antibiotic agar plates.

### Nucleosome sequence design

Nucleosome positions in PHO5pr were estimated based on a nucleosome position database [33]. The GC% of wild-type nucleosome -2 sequence (without the high affinity Pho4 binding site) is 39%. We varied GC% around this value and confirmed whether the in vivo nucleosome preference changed as predicted based on a published model [30]. Changing the nucleosome -2 sequence may introduce additional transcription factor binding sites that can affect gene expression level. Hence, all sequences were checked for the absence of Pho4 and any other additional transcription factor binding sites relative to the 39% sequence using an online tool [49]. It was not possible, however, to avoid an additional Stb5 binding site for sequences for GC% higher than 50%. The sequences are given in Table S3.

### Fluorescence measurements

For single-cell fluorescence measurements, cells were grown to saturation overnight at 30°C in 3 ml synthetic complete medium. Overnight cultures were sub-inoculated in a new 3 ml medium and grown for about 9–10 hours to reach an optical density at 600 nm (OD_600_) of about 0.5. Each culture was further diluted by transferring to 3 ml media containing doxycycline at concentrations 0, 0.1, 0.2, 0.4 and 0.8 µg/ml and grown for another 16 hours. The measured optical densities were about 0.5 for all cultures at this point. The samples were prepared for microscopy as described previously [15]. Fluorescence microscopy was performed on a motorized inverted microscope (IX81; Olympus) with an interline CCD (Clara; Andor Technology) and an Argon-Krypton laser (LS300; Dynamic laser). Fluorescence band-pass filters were used for the detection of CFP (FF01-482/25-25; Semrock), YFP (FF01-535/22) and RFP (FF01-609/54-25). The acquisition routine was automated using Micro-Manager Open Source Software [50]. Single-cell images from each channel were analyzed and processed using an in-house Matlab program. To define background levels of YFP and CFP for different promoter variants, we added reference cells without YFP or CFP for every GRF measurement.

### Chromatin immunoprecipitation

Chromatin immunoprecipitation (ChIP) was performed as described [51] with the following modifications. All strains were grown up to 0.6 OD_600_ and cross-linked in 1% formaldehyde for 15 min at room temperature with occasional swirling of culture flasks. Cross-linking was stopped by adding 0.125 M heat-sterilized glycine into the cultures and 5 min incubation at room temperature. Cells were then harvested and washed twice with ice-cold TBS buffer and once with ice-cold lysis buffer (50 mM HEPES-KOH, pH 7.5, 150 mM NaCl, 1% Triton X-100, 0.1% sodium deoxycholate). Harvested cells were then disrupted with glass beads in the lysis buffer with 2 mM PMSF at 4°C by vortexing on high speed for 2 hours. Disrupted cells were then separated from glass beads, and cell lysate was prepared by centrifuging them at high speed at 4°C. Resulting chromatin was sheared by sonication using a micro tip (Sonic ruptor 250, Omni international) with 20% output power and continuous pulse for 30 seconds four times. From the total sheared chromatin lysate, 10% was set aside as a normalization control (input DNA). The rest of the sample was incubated with 2.5 µg of antibody against the C-terminus of human histone H3 (abcam) overnight at 4°C. The antibody-histone complex was precipitated using Pierce Agarose ChIP kit (Thermo Scientific) following manufacturer’s instructions. The resulting immunoprepitated DNA (IP DNA) was quantified by real-time PCR.

### In vivo nucleosome mapping

In-vivo nucleosome mapping was performed by quantitative real-time PCR (qPCR) of IP DNA using SsoFast EvaGreen Supermix (Bio-Rad Laboratories). We used five primer pairs targeting the PHO5pr region from nucleosome -3 to nucleosome -1 and one primer pair targeting the control locus REC104 for this analysis (Table S4). To obtain the relative nucleosome occupancy, we generated the standard curve from serially diluted input DNA for all targets, quantified the amount of IP DNA using the standard curve, and divided this amount by the amount of the control IP DNA (REC 104).

## Supporting Information

Figure S1 **In-vivo nucleosome occupancy maps of the promoter variant without nucleosome -2 (Nuc -2 (−)).** Relative nucleosome occupancies (in arbitrary units) were measured by quantitative ChIP at three different promoter locations (in base pairs). The nucleosome occupancy at REC104 locus was used as the reference value. The error bars represent the standard deviation of three independent measurements. Genomic coordinates of nucleosome positions (top schematic) were obtained from a compiled database for nucleosome positioning in *S. cerevisiae* [33].
(TIF)

Figure S2 **Nonequilibrium model of gene expression.** The red arrows are reactions that depend on nucleosome stability. The irreversible chromatin remodeling step represented by *k_r_* brings (1,1,1) back to (1,0,0), and completes the steady-state cycle.
(TIF)

Figure S3 **Predicted GRF.** The model explains the non-monotonic change in the flux as a function of transcription factor input and as a function of nucleosome occupancy (*f* ). Case 1 and Case 2 show the asymmetry of the predicted GRF with respect to *p_1_* and *p_2_*. Case 1 is for *p_1_*<*p_2_*, and Case 2 is for *p_1_*>*p_2_*.
(TIF)

Figure S4 **Fitting of GRFs. Here, the GRF is generated using a slightly different binning method than in** **Figure 3** **.** To generate the average GRF, CFP (output) intensity values were plotted against the common logarithm (log_10_) of corresponding YFP (input) intensity values. The x-data, equal to log_10_ (YFP intensity), were binned with a variable bin-width so that each bin contains the same number of data points (the bin intervals are quantiles of the x-data). CFP and YFP intensity values within each bin were averaged, and the mean values are plotted as circles. Each GRF is fit independently with four coefficients according to Equation S12. *p_4_* was constrained to be smaller than 1. (*p_1_*, *p_2_*, *p_3_*, *p_4_*) are (315, 936, 115, 1.00) for 24%, (124, 10642, 785, 0.92) for 39%, (179, 18180, 440, 0.99) for 54%, respectively.
(TIF)

Figure S5 **The nonequilibrium flux.** The system can be driven out of equilibrium by dialing up the maximum remodeling rate (*k*). The flux vs. transcription factor input when *k* = 1 is plotted on the left with three different nucleosome occupancy values (*f* = 0.1, 0.5, and 0.75), and they closely resemble the near-equilibrium curves shown in Figure S3. On the right, the flux at a fixed transcription factor input is plotted as a function of the maximum remodeling rate (*k*). Beyond the crossover point between *f = *0.5 and *f* = 0.75, the dependence of flux on nucleosome occupancy becomes monotonic.
(TIF)

Table S1 **Equilibrium probability of each promoter state.** The promoter state is defined by the occupancy of the respective sites on DNA (*D*) by transcription factor (*T*), chromatin remodeling complex (*C*), and the nucleosome (*N*). ‘[ ]’ denotes concentration. *AB* indicates the complex formed between A and B. The interaction can be described by the interaction energy ε_AB_ or the equilibrium dissociation constant *K_AB_.c_0_* is the reference concentration at standard state that relates the two. The expressions marked in red are the approximations used for this study.
(DOCX)

Table S2 **The fitting parameters from our quantitative model.**
(DOCX)

Table S3 **147 bp DNA sequences used to change stability of nucleosome -2.**
(DOCX)

Table S4 **Primers used for quantitative real-time PCR.**
(DOCX)

Text S1 **Detailed description of the quantitative model.**
(DOCX)
