# Supplementary figures and images for: The Effect of an Intervening Promoter Nucleosome on Gene Expression
Source: PLoS One. 2013 May 20;8(5):e63072. doi: 10.1371/journal.pone.0063072 (PMC3659125; doi:10.1371/journal.pone.0063072)

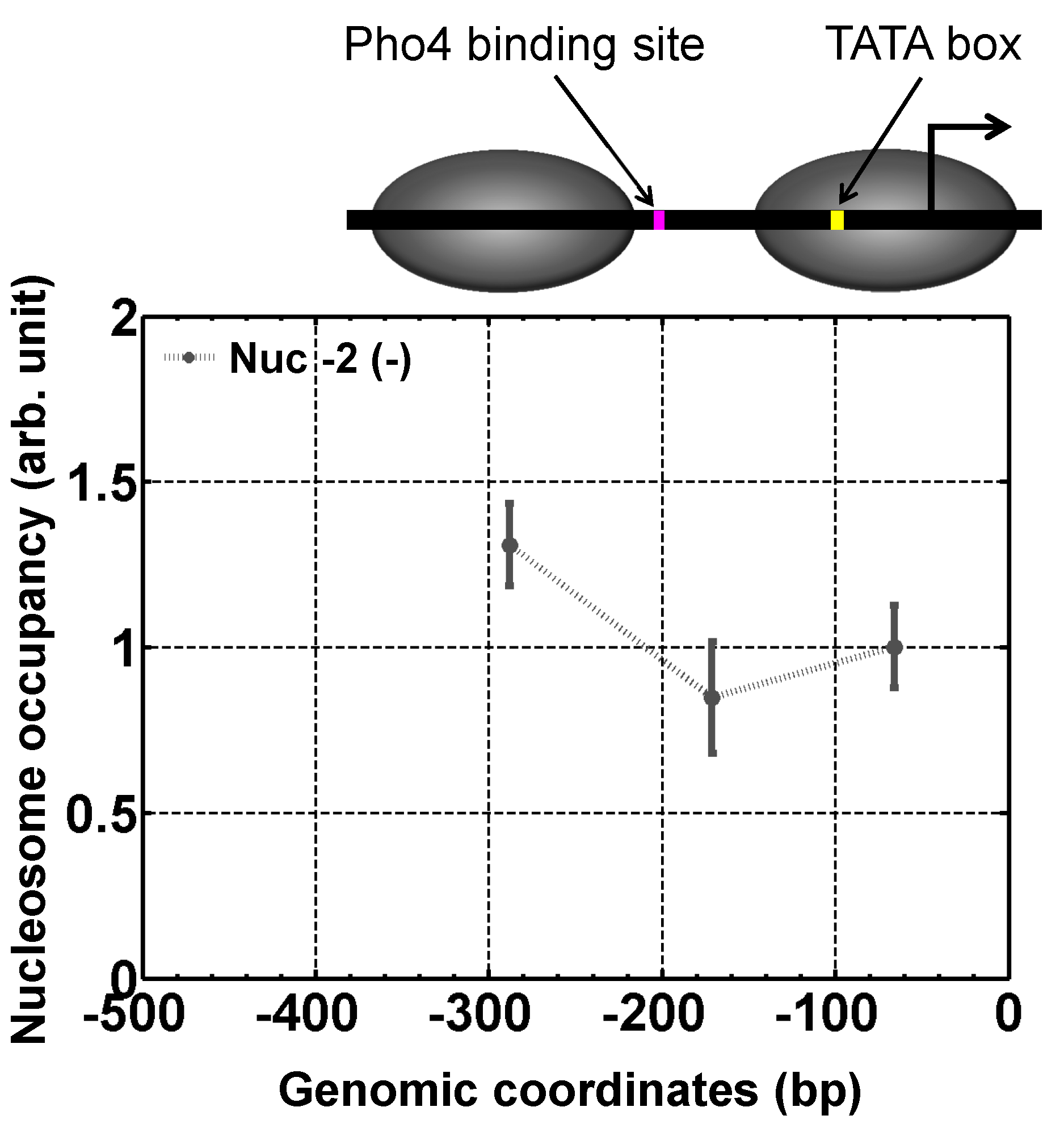

Supplement: Figure S1 — In-vivo nucleosome occupancy maps of the promoter variant without nucleosome -2 (Nuc -2 (−)). Relative nucleosome occupancies (in arbitrary units) were measured by quantitative ChIP at three different promoter locations (in base pairs). The nucleosome occupancy at REC104 locus was used as the reference value. The error bars represent the standard deviation of three independent measurements. Genomic coordinates of nucleosome positions (top schematic) were obtained from a compiled database for nucleosome positioning in S. cerevisiae [33]. (TIF) [file pone.0063072.s001.tif]

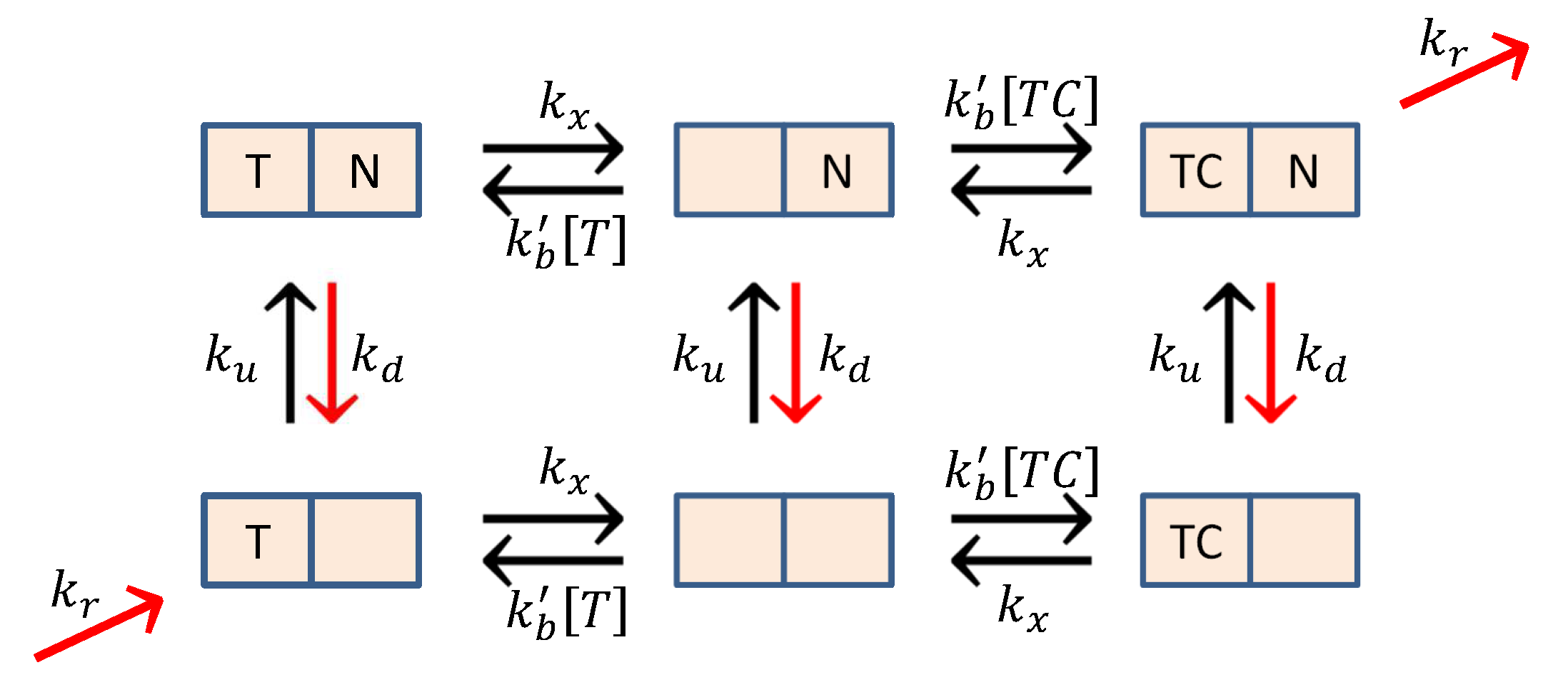

Supplement: Figure S2 — Nonequilibrium model of gene expression. The red arrows are reactions that depend on nucleosome stability. The irreversible chromatin remodeling step represented by kr brings (1,1,1) back to (1,0,0), and completes the steady-state cycle. (TIF) [file pone.0063072.s002.tif]

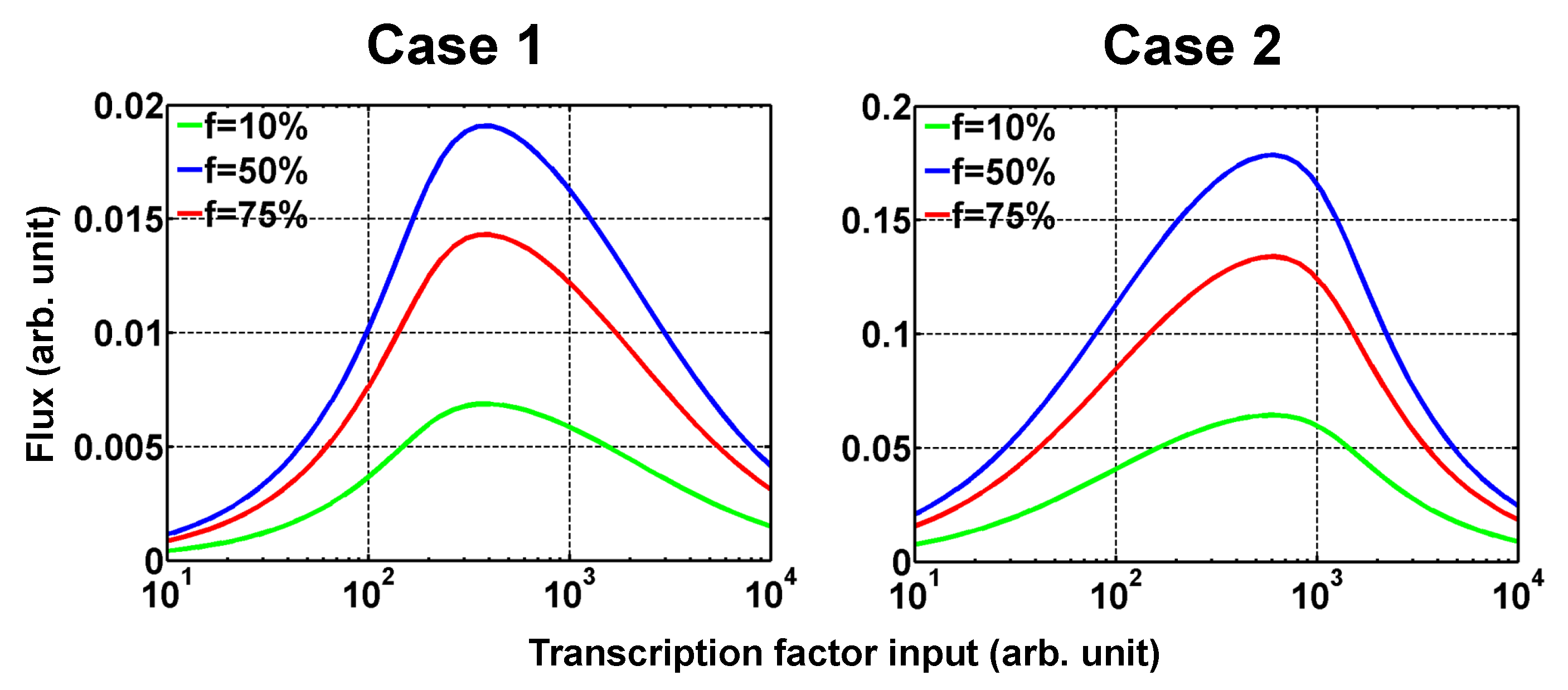

Supplement: Figure S3 — Predicted GRF. The model explains the non-monotonic change in the flux as a function of transcription factor input and as a function of nucleosome occupancy (f ). Case 1 and Case 2 show the asymmetry of the predicted GRF with respect to p1 and p2. Case 1 is for p1<p2, and Case 2 is for p1>p2. (TIF) [file pone.0063072.s003.tif]

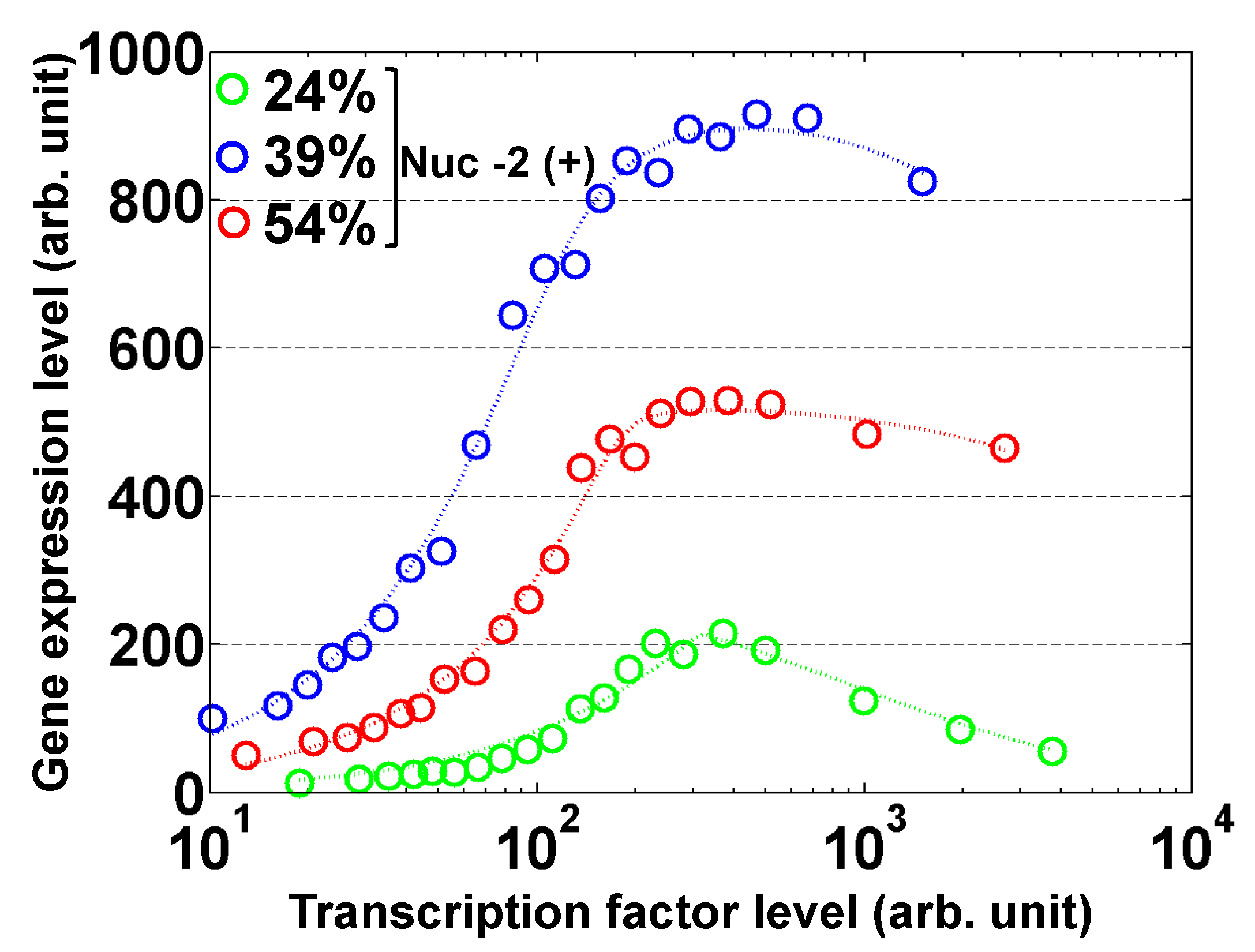

Supplement: Figure S4 — Fitting of GRFs. Here, the GRF is generated using a slightly different binning method than in Figure 3 . To generate the average GRF, CFP (output) intensity values were plotted against the common logarithm (log10) of corresponding YFP (input) intensity values. The x-data, equal to log10 (YFP intensity), were binned with a variable bin-width so that each bin contains the same number of data points (the bin intervals are quantiles of the x-data). CFP and YFP intensity values within each bin were averaged, and the mean values are plotted as circles. Each GRF is fit independently with four coefficients according to Equation S12. p4 was constrained to be smaller than 1. (p1, p2, p3, p4) are (315, 936, 115, 1.00) for 24%, (124, 10642, 785, 0.92) for 39%, (179, 18180, 440, 0.99) for 54%, respectively. (TIF) [file pone.0063072.s004.tif]

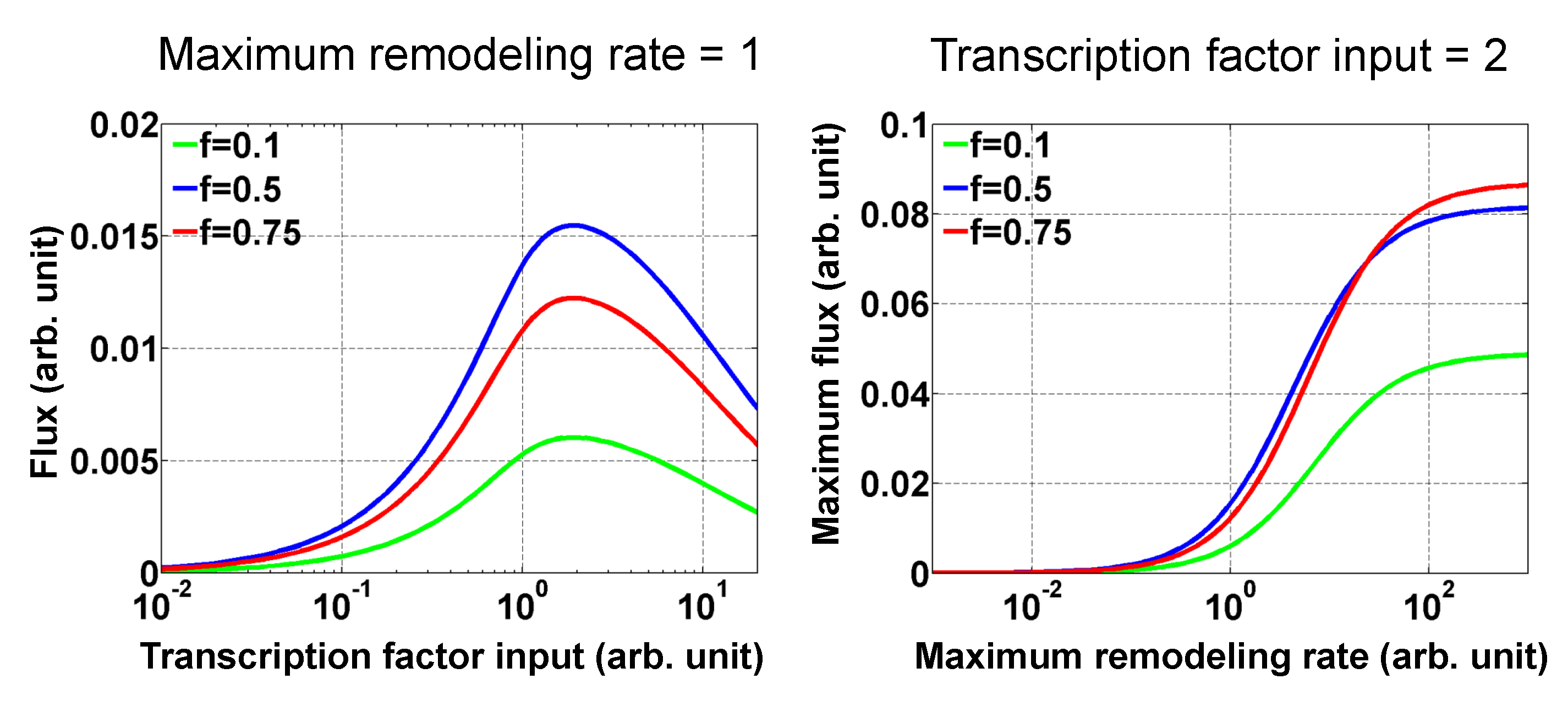

Supplement: Figure S5 — The nonequilibrium flux. The system can be driven out of equilibrium by dialing up the maximum remodeling rate (k). The flux vs. transcription factor input when k = 1 is plotted on the left with three different nucleosome occupancy values (f = 0.1, 0.5, and 0.75), and they closely resemble the near-equilibrium curves shown in Figure S3. On the right, the flux at a fixed transcription factor input is plotted as a function of the maximum remodeling rate (k). Beyond the crossover point between f = 0.5 and f = 0.75, the dependence of flux on nucleosome occupancy becomes monotonic. (TIF) [file pone.0063072.s005.tif]
