# Supplementary material for: The Effect of an Intervening Promoter Nucleosome on Gene Expression
Source: PLoS One. 2013 May 20;8(5):e63072. doi: 10.1371/journal.pone.0063072 (PMC3659125; doi:10.1371/journal.pone.0063072)
Supplement: Table S1 — Equilibrium probability of each promoter state. The promoter state is defined by the occupancy of the respective sites on DNA (D) by transcription factor (T), chromatin remodeling complex (C), and the nucleosome (N). ‘[ ]’ denotes concentration. AB indicates the complex formed between A and B. The interaction can be described by the interaction energy εAB or the equilibrium dissociation constant KAB.c0 is the reference concentration at standard state that relates the two. The expressions marked in red are the approximations used for this study. (DOCX) [file pone.0063072.s006.docx]

| Promoter state (T,C,N) | Relative occupancy |
| --- | --- |
| (0,0,0) | $\left[ D \right]$ |
| (1,0,0) | $\left[ DT \right]=\frac{\left[ D \right]\left[ T \right]}{c_{0}}e^{-\varepsilon_{DT}}=\frac{\left[ D \right]\left[ T \right]}{K_{DT}}$ |
| (0,1,0) | $\left[ DC \right]=\frac{\left[ D \right]\left[ C \right]}{c_{0}}e^{-\varepsilon_{DC}}=\frac{\left[ D \right]\left[ C \right]}{K_{DC}}\ll\left[ D \right]$ |
| (0,0,1) | $\left[ DN \right]=\frac{\left[ D \right]\left[ N \right]}{c_{0}}e^{-\varepsilon_{DN}}=\frac{\left[ D \right]}{K_{DN}}$ |
| (1,1,0) | $\left[ DTC \right]=\frac{\left[ D \right]\left[ T \right]\left[ C \right]}{c_{0}^{2}}e^{-\left( \varepsilon_{DT}+\varepsilon_{DC}+\varepsilon_{TC} \right)}\approx\frac{\left[ D \right]\left[ T \right]\left[ C \right]}{c_{0}^{2}}e^{-\left( \varepsilon_{DT}+\varepsilon_{TC} \right)}=\frac{\left[ D \right]\left[ TC \right]}{K_{DT}}$ |
| (1,0,1) | $\left[ DTN \right]=\frac{\left[ D \right]\left[ T \right]\left[ N \right]}{c_{0}^{2}}e^{-\left( \varepsilon_{DT}+\varepsilon_{DN} \right)}=\frac{\left[ D \right]\left[ T \right]}{K_{DT}K_{DN}}$ |
| (0,1,1) | $\left[ DCN \right]=\frac{\left[ D \right]\left[ C \right]\left[ N \right]}{c_{0}^{2}}e^{-\left( \varepsilon_{DN}+\varepsilon_{DC}+\varepsilon_{CN} \right)}=\frac{\left[ DN \right]\left[ C \right]}{K_{CN}}\ll\left[ DN \right]$ |
| (1,1,1) | $\left[ DTCN \right]\approx\frac{\left[ D \right]\left[ T \right]\left[ C \right]\left[ N \right]}{c_{0}^{3}}e^{-\left( \varepsilon_{DT}+\varepsilon_{DN}+\varepsilon_{TC}+\varepsilon_{CN} \right)}=\frac{\left[ D \right]\left[ TC \right]}{K_{DT}K_{DN}}e^{-\varepsilon_{CN}}\approx\frac{\left[ D \right]\left[ TC \right]}{K_{DT}K_{DN}}$ |
