# Supplementary material for: The Effect of an Intervening Promoter Nucleosome on Gene Expression
Source: PLoS One. 2013 May 20;8(5):e63072. doi: 10.1371/journal.pone.0063072 (PMC3659125; doi:10.1371/journal.pone.0063072)
Supplement: Table S2 — The fitting parameters from our quantitative model. (DOCX) [file pone.0063072.s007.docx]

| Parameter | Explicit meaning | Implicit meaning |
| --- | --- | --- |
| $p_{1}$ | Rise/fall threshold | $\left[ C \right]_{tot}+K_{TC}$ |
| $p_{2}$ | Fall/rise threshold | $K_{DT}$ |
| $p_{3}$ | Maximum value | $\frac{k\left[ C \right]_{tot}K_{DN}}{\left( \sqrt{\left[ C \right]_{tot}+K_{TC}}+\sqrt{K_{DT}} \right)^{2}\left( K_{DN}+K_{M} \right)\left( K_{DN}+1 \right)}$ |
| $p_{4}$ | Skewness | $\frac{\left[ C \right]_{tot}}{\left[ C \right]_{tot}+K_{TC}}$ |
