# Supplementary material for: The Effect of an Intervening Promoter Nucleosome on Gene Expression
Source: PLoS One. 2013 May 20;8(5):e63072. doi: 10.1371/journal.pone.0063072 (PMC3659125; doi:10.1371/journal.pone.0063072)
Supplement: Table S3 — 147 bp DNA sequences used to change stability of nucleosome -2. (DOCX) [file pone.0063072.s008.docx]

| GC% | DNA sequence (5′→3′) |
| --- | --- |
| 24 | ATAGTTAATTAAATAGTAAATCTTTAAATGAATAGATACAACCTTGGCACTCAAATTTTGGAATATTACAGACTAAATTTATAATTATATTACTTGTTTTCGAAAAGATCGCAAATGTCAAATTATCAAATTGTTCAACTTACTTGG |
| 39 | AGAGTTAATTGAATAGGCAATCTCTAAATGAATCGATACAACCTTGGCACTCAAAGCTTGGACTAGCACAGACTAAATTTATGATTCTGGTCCCTGTTTTCGAAGAGATCGCACATGCCAAATTATCAAATTGGTCACCTTACTTGG |
| 54 | AGAGTGAATTGAGTAGGCAGCCTCTAAACGCATCGACACAGCCGTGGCACTCAAAGCTTGGACTAGCGCAGCCTGAACGTACGATTCTGGTCCCCGTTTTCGAAGCGATCGCGCGTGCCAAGTCATCACATTGGTCACCTTACTTGG |
