# Supplementary material for: The Effect of an Intervening Promoter Nucleosome on Gene Expression
Source: PLoS One. 2013 May 20;8(5):e63072. doi: 10.1371/journal.pone.0063072 (PMC3659125; doi:10.1371/journal.pone.0063072)
Supplement: Table S4 — Primers used for quantitative real-time PCR. (DOCX) [file pone.0063072.s009.docx]

| Target | Strain | Primer Sequence (5′→3′) | |
| --- | --- | --- | --- |
| Nucleosome -2 | 24% | f | AAATGAATAGATACAACCTTGGCACT |
|  |  | r | TGCGATCTTTTCGAAAACAAG |
|  | 39% | f | TCTAAATGAATCGATACAACCTTGG |
|  |  | r | CTTCGAAAACAGGGACCAGA |
|  | 54% | f | GCCTCTAAACGCATCGACAC |
|  |  | r | AAACGGGGACCAGAATCG |
| Linker 1 | All | f | GCTTTTTCTTTGTCTGCACAAAG |
|  | Nucleosome (-) | r | ACGTTTCATTTCGACAATTCAAA |
|  | 24% | r | AGTGCCAAGGTTGTATCTATTCATTT |
|  | 39% | r | CCAAGGTTGTATCGATTCATTTAGA |
|  | 54% | r | GTGTCGATGCGTTTAGAGGC |
| Linker 2 | 24% | f | CTTGTTTTCGAAAAGATCGCA |
|  | 39% | f | TCTGGTCCCTGTTTTCGAAG |
|  | 54% | f | CGATTCTGGTCCCCGTTT |
|  | All | r | ACGTTTCATTTCGACAATTCAAA |
| Nucleosome -1 | All | f | TTTGAATTGTCGAAATGAAACGT |
|  |  | r | TCGAATTTGCTTGCTCTATTTGT |
| Nucleosome -3 | All | f | AATGCGCAAATATGTCAACGT |
|  |  | r | CTTTGTGCAGACAAAGAAAAAGC |
| REC 104 | All | f | CCTTTAGCTAATAGAGTAAGCCACA |
|  |  | r | TTTAACACTACTGGTTTATGAAAGAAA |
