# Supplementary material for: The Effect of an Intervening Promoter Nucleosome on Gene Expression
Source: PLoS One. 2013 May 20;8(5):e63072. doi: 10.1371/journal.pone.0063072 (PMC3659125; doi:10.1371/journal.pone.0063072)
Supplement: Text S1 — Detailed description of the quantitative model. (DOCX) [file pone.0063072.s010.docx]

# Supporting Information

# The Non-monotonic Effect of Nucleosome Occupancy on Gene Expression

Rasesh Y. Parikh & Harold D. Kim

**Quantitative model**

The quantitative model for the gene regulation function (GRF) presented in the main text combines promoter transitions with molecular mechanism of chromatin remodeling. Promoter transitions are treated as reversible, and the chromatin remodeling step irreversible. The system is an open system where there is a continuous turnover of nucleosomes and ATP, and therefore the detailed balance does not hold. This aspect is missing in other gene regulation models where the promoter is treated to be in equilibrium with energy-independent transcription factors only [1].

Another important aspect of this quantitative model is that we focus on the fate of the first nucleosome only. The state of the second nucleosome can be included in the model as in our previous description [2], but here we argue that based on the mechanism of chromatin remodeling, transcriptional activity is determined mostly by the fate of the first nucleosome. There are eight different promoter states based on the occupancy of transcription factor (T), chromatin remodeling complex (C) and the nucleosome (N). We use (T,C,N) to denote the promoter state where T, C, and N can be either zero or one depending on its occupancy. Among these eight states, we omitted (0,1,0) and (0,1,1) because the chromatin remodeling complex SWI/SNF is recruited to PHO5pr by the transcription factor Pho4 and is unlikely to find PHO5pr by itself. We reason that (1,1,1) is primed for chromatin remodeling whereas (1,1,0) is not.

We also assume that the binding rates are of first order with respect to the transcription factors, and the unbinding rates are constant, determined only by the interaction between DNA and the transcription factor. We thus have five rate constants for reversible reactions (Figure S2): $k_{u}$and $k_{d}$ for the upward and downward transitions that determine nucleosome stability, $k_{b}'\left[T\right]$ for binding of T to DNA, $k_{b}'\left[TC\right]$for binding of TC to DNA, and $k_{x}$ for unbinding of T and TC from DNA. Once the promoter state reaches (1,1,1), the nucleosome is efficiently translocated towards the TATA box, thus causing the removal of other nucleosomes along its way. This ATP-dependent, irreversible step brings (1,1,1) back to (1,0,0). Hence, we assign a unidirectional arrow to this transition, which keeps the promoter out of thermodynamic equilibrium.

As discussed in the main text, we assume that this chromatin remodeling step depends on the nucleosome stability: a more stable nucleosome is more difficult to be translocated by the chromatin remodeling complex. The Brownian ratchet mechanism provides a simple way to incorporate this dependence into the chromatin remodeling rate. In this model, DNA can spontaneously unwrap from the histone octamer for a transient period, during which the chromatin remodeling complex inches forward. The net rate of translocation will increase with the unwrapping rate of DNA and reach the maximum rate at which the chromatin remodeling complex can move unobstructed as on a free DNA. Because the unwrapping rate of DNA is proportional to the nucleosome dissociation rate ($k_{d}$), the dependence of the remodeling rate ($k_{r}$) on $k_{d}$ is given by a Michaelis-Menten type function:

| $k_{r}=\frac{kk_{d}}{k_{d}+k_{m}}$ | Equation S1 |
| --- | --- |

Where $k_{m}$ is the midpoint stability that leads to the half-maximum remodeling rate.

In this nonequilibrium model, the transcription rate depends on the flux J from (1,1,1) to (1,0,0), which is the product of the steady-state probability of (1,1,1) and the remodeling rate:

| $J=P_{\left(1,1,1\right)}k_{r\cdot}$ | Equation S2 |
| --- | --- |

It should be noted that the steady-state probabilities cannot be obtained from the detailed balance condition because the system is not in thermodynamic equilibrium. We used the Symbolic Math Toolbox in Matlab to find the steady-state current. However, the solution contains more than hundred terms, and is difficult to characterize. Therefore, we solved for the solution in the equilibrium limit ($k_{r}\to0$) and slowly increase $k_{r}$ to examine $J$ in the nonequilibrium regime.

# Derivation of the fitting function from near-equilibrium flux

The flux near equilibrium can be obtained by applying the detailed balance condition. All pairwise reversible reactions are considered to be in equilibrium, and therefore individual rate constants are not necessary, and equilibrium constants are sufficient to describe the steady state fraction of each state as listed in Table S1. To reduce the number of fitting parameters, we ignored the interaction between the chromatin remodeling complex and the nucleosome. According to Equation S1 and Equation S2, the flux is

| $J\approx\frac{kK_{DN}}{\left(K_{DN}+K_{M}\right)\left(K_{DN}+1\right)}\frac{\left[TC\right]}{\left(\left[T\right]_{tot}+K_{DT}\right)}\cdot$ | Equation S3 |
| --- | --- |

Here, $K_{DT}$is the equilibrium dissociation constant for the interaction between DNA and the transcription factor ($K_{DT}=k_{x}/k_{b}'$), and $K_{DN}$ for the interaction between DNA and the histone octamer ($K_{DN}=k_{d}/k_{u}$). $K_{M}$ is the ratio of $k_{m}$ to $k_{u}$. $\left[T\right]_{tot}$ is the total concentration of the transcription factor which is the actual measurable via YFP fluorescence in our experiment. Thus, we express the equilibrium concentrations of $\left[TC\right]$ and $\left[T\right]$ in terms of $\left[T\right]_{tot}$ using the equilibrium dissociation constant$K_{TC}$:

| $\frac{\left(\left[T\right]_{tot}-\left[TC\right]\right)\left(\left[C\right]_{tot}-\left[TC\right]\right)}{\left[TC\right]}=K_{TC}\cdot$ | Equation S4 |
| --- | --- |

Solving for$\left[TC\right]$,

| $\left[TC\right]=\frac{1}{2}\left[\left(\left[T\right]_{tot}+\left[C\right]_{tot}+K_{TC}\right)-\sqrt{\left(\left[T\right]_{tot}+\left[C\right]_{tot}+K_{TC}\right)^{2}-4\left[C\right]_{tot}\left[T\right]_{tot}}\right]\cdot$ | Equation S5 |
| --- | --- |

Substituting Equation S5 into Equation S3, we can obtain the flux as a function of $\left[T\right]_{tot}$.

| $J\approx\frac{kK_{DN}}{2\left(K_{DN}+K_{M}\right)\left(K_{DN}+1\right)\left(\left[T\right]_{tot}+K_{DT}\right)}\left[\begin{matrix} \left(\left[T\right]_{tot}+\left[C\right]_{tot}+K_{TC}\right) \\ -\sqrt{\left(\left[T\right]_{tot}+\left[C\right]_{tot}+K_{TC}\right)^{2}-4\left[C\right]_{tot}\left[T\right]_{tot}} \end{matrix}\right]\cdot$ | Equation S6 |
| --- | --- |

Assuming that the measured gene expression level ($y$) is proportional to the flux, the gene regulation function (GRF) can be fit with four independent parameters,$A$, $K_{DT}$, $\left[C\right]_{tot}$, and$K_{TC}$:

| $y=\frac{A}{\left(x+K_{DT}\right)}\left[\left(x+\left[C\right]_{tot}+K_{TC}\right)-\sqrt{\left(x+\left[C\right]_{tot}+K_{TC}\right)^{2}-4\left[C\right]_{tot}x}\right],$ | Equation S7 |
| --- | --- |

where $x$ represents the measured transcription factor level in arbitrary units, and$K_{DT}$, $\left[C\right]_{tot}$, and $K_{TC}$ are normalized in the same dimension as$x$.

In the current form, it is difficult to relate these parameters to the visual characteristics of this function. Therefore, we approximate Equation S7 as

| $y\approx\frac{A}{x+K_{DT}}\cdot\frac{2\left[C\right]_{tot}x}{x+\left[C\right]_{tot}+K_{TC}}\cdot$ | Equation S8 |
| --- | --- |

This function can be decomposed into two logistic functions:

| $y\approx\frac{Bx}{\left(x+p_{1}\right)\left(x+p_{2}\right)}=\frac{B}{p_{2}-p_{1}}\left(\frac{x}{x+p_{1}}-\frac{x}{x+p_{2}}\right),$ | Equation S9 |
| --- | --- |

where we changed the variables for convenience of interpretation. If $p_{2}>p_{1}$, the first logistic function rises around$p_{1}$, and the second logistic function falls around $p_{2}$. Therefore, these fitting parameters can be understood as the rise threshold and the fall threshold of GRF, respectively. One can further show that $y$ has a maximum when $x=\sqrt{p_{1}p_{2}}$:

| $y\approx\frac{B}{x+\frac{p_{1}p_{2}}{x}+p_{1}+p_{2}}\leq\frac{B}{\left(\sqrt{p_{1}}+\sqrt{p_{2}}\right)^{2}}\cdot$ | Equation S10 |
| --- | --- |

Using the maximum value as the third fitting parameter $p_{3}$ instead of $B$, we can obtain an expression which includes these three parameters that can be easily identified from the curve.

| $y\approx\frac{p_{3}\left(\sqrt{p_{1}}+\sqrt{p_{2}}\right)^{2}x}{\left(x+p_{1}\right)\left(x+p_{2}\right)},$ | Equation S11 |
| --- | --- |

Now that we have gained insight into some of the parameters, we can go back to the original expression (Equation S6) and adjust the remaining parameters. The smaller of $K_{DT}$ and $\left[C\right]_{tot}+K_{TC}$ becomes $p_{1}$, and the larger becomes$p_{2}$. We also define the fourth parameter $p_{4}$, a proper fraction ($0<p_{4}<1$) that represents the skewness of the curve. Assuming $K_{DT}$ is the larger of the two,

| $y=\frac{p_{3}\left(\sqrt{p_{1}}+\sqrt{p_{2}}\right)^{2}}{2p_{1}p_{4}\left(x+p_{2}\right)}\left[\left(x+p_{1}\right)-\sqrt{\left(x+p_{1}\right)^{2}-4p_{1}p_{4}x}\right]\cdot$ | Equation S12 |
| --- | --- |

The dependence of these four fitting parameters on the model parameters is summarized in Table S2 .

The only parameter that depends on $K_{DN}$ is $p_{3}$, which represents the maximum expression level of the GRF. The dependence of $p_{3}$ on $K_{DN}$ is virtually identical to Equation S9:

| $p_{3}\propto\frac{K_{DN}}{\left(K_{DN}+K_{M}\right)\left(K_{DN}+1\right)}\cdot$ | Equation S13 |
| --- | --- |

Hence, $p_{3}$ can acquire a non-monotonic dependence on the nucleosome stability. To illustrate this point, we consider the case of $K_{M}=1$ where $p_{3}$ is most sensitive to the nucleosome stability. In this case, $p_{3}$ is proportional to $f\left(1-f\right)$, where$f$ is the nucleosome occupancy. The GRF (Equation S12) is plotted in Figure S3 with three different $f$ values. In our global fitting of GRFs of three different GC contents, we let only $p_{3}$ vary while keeping $p_{1}$, $p_{2}$, and $p_{4}$ the same among them. The result of this global fitting is presented in the main text (Figure 3). We also attempted to fit each GRF individually with the four fitting parameters. In this fitting, all four parameters depend on the nucleosome stability. Equation S12 fits all GRFs exceptionally well (Figure S4).

# Comment on thresholds

Although the approximate expression (Equation S11) for the GRF is symmetric between$p_{1}$ and $p_{2}$, the exact form (Equation S12) is not. Therefore, the shape of the curve changes depending on which of$p_{1}$ and $p_{2}$ is larger. $K_{DN}$ is on the order of 10 nM based on an in vitro measurement [3]. It must be noted that the in vitro measurement focuses on one specific binding site of DNA and the protein, but in vivo, there are numerous identical sites across the genome, which is estimated to be on the order of hundreds. This effectively reduces the binding affinity to the particular site of PHO5pr about ~100-fold. SWI/SNF is a low abundance protein with ~100-200 copies per cell [4,5] and participates in transcription of 6% of the genes in yeast [6]. It is also shared by other transactivators such as Gcn4, Hap4, Gal4, and Swi5 [7]. These complications make it difficult to compare $\left[C\right]_{tot}+K_{TC}$ and $K_{DT}$, and therefore we consider both cases.

Case 1: $\left[C\right]_{tot}+K_{TC}<K_{DT}$, ($\left[C\right]_{tot}=200,K_{TC}=20,K_{DT}=2000$)

Case 2: $\left[C\right]_{tot}+K_{TC}>K_{DT}$, ($\left[C\right]_{tot}=1000,K_{TC}=100,K_{DT}=100$)

Because the measured GRF is heavily skewed towards one direction on the log axis, we keep $\left[C\right]_{tot}$ significantly larger than $K_{TC}$ so that $p_{4}$ is closer to one. Case 1 better reproduces the observed skew of the GRF: it rises steeply and decays gradually.

# The behavior of nonequilibrium steady state flux

The nonequilibrium flux depends on a larger number of parameters than the equilibrium flux, and therefore, can certainly describe the behavior of the measured GRFs. Instead of performing a global fitting with a larger number of fitting parameters, we asked how much the steady state flux deviates from the equilibrium flux if the system is driven further away from equilibrium. Using a set of parameters that closely reproduced the measured GRFs in near equilibrium, we examined how the steady state flux changes with increasing $k$ (maximum remodeling rate), especially focusing on the extent for which the non-monotonic dependence of flux ($J$) on nucleosome occupancy ($f$) is preserved. We found that the nonequilibrium flux pattern closely resembles the equilibrium flux pattern over a wide range of $k$ (Figure S5). When $k$ exceeds the unbinding rate constant ($k_{x}$), which is the fastest rate constant of the system, the non-monotonic pattern breaks down (Figure S5). As shown, the flux at a fixed transcription factor input level increases as a function of the maximum remodeling rate in a logistic fashion, which is a general behavior of biological systems in nonequilibrium [8].

**References**

1. Sanchez A, Garcia HG, Jones D, Phillips R, Kondev J (2011) Effect of Promoter Architecture on the Cell-to-Cell Variability in Gene Expression. PLoS Comput Biol 7: e1001100. doi:10.1371/journal.pcbi.1001100.

2. Kim HD, O’Shea EK (2008) A quantitative model of transcription factor-activated gene expression. Nat Struct Mol Biol 15: 1192–1198. Available: http://dx.doi.org/10.1038/nsmb.1500.

3. Maerkl SJ, Quake SR (2007) A Systems Approach to Measuring the Binding Energy Landscapes of Transcription Factors. Science 315: 233–237. doi:10.1126/science.1131007.

4. Cote J, Quinn J, Workman JL, Peterson CL (1994) Stimulation of GAL4 derivative binding to nucleosomal DNA by the yeast SWI/SNF complex. Science 265: 53–60. doi:10.1126/science.8016655.

5. Peterson CL, Workman JL (2000) Promoter targeting and chromatin remodeling by the SWI/SNF complex. Current Opinion in Genetics & Development 10: 187–192. doi:10.1016/S0959-437X(00)00068-X.

6. Sudarsanam P., Winston F. (2000) The Swi/Snf family - nucleosome-remodeling complexes and transcriptional control. Trends in Genetics 16: 345–351. doi:10.1016/S0168-9525(00)02060-6.

7. Prochasson P, Neely KE, Hassan AH, Li B, Workman JL (2003) Targeting Activity Is Required for SWI/SNF Function In Vivo and Is Accomplished through Two Partially Redundant Activator-Interaction Domains. Molecular Cell 12: 983–990. doi:10.1016/S1097-2765(03)00366-6.

8. Rothschild KJ, Ellias SA, Essig A, Stanley HE (1980) Nonequilibrium linear behavior of biological systems. Existence of enzyme-mediated multidimensional inflection points. Biophysical Journal 30: 209–230. doi:10.1016/S0006-3495(80)85090-9.
